# Stevens-Johnson syndrome after complete recovery of pneumonia: a clinical image

**DOI:** 10.11604/pamj.2022.43.170.38049

**Published:** 2022-12-05

**Authors:** Zothanpuii Ralte, Trupti Uke

**Affiliations:** 1 Department of Mental Health Nursing, Smt Radhikabai Meghe Memorial College of Nursing, Datta Meghe Institute of Medical Sciences, Sawangi, Wardha, Maharashtra, India

**Keywords:** Stevens-Johnson syndrome, lesions, peri orbital edema, rash, blisters

## Image in medicine

Stevens-Johnson syndrome (SJS) is a rare, serious disorder of the skin and mucous membranes. It's usually a reaction to medication that starts with flu-like symptoms, followed by a painful rash that spreads and blisters. Then the top layer of affected skin dies, sheds and begins to heal after several days. Here we are presenting a case of an 11-month-old male child with complaints of dry, scaly lesions all over the body along with fever for 5 days. The lesions started initially on the cheeks and gradually covered the body the next day. The lesions covered both the eyes and caused peri orbital edema. He has history of pneumonia 2 months back, in which he was treated with antibiotic. The baby was started with injection of Augmentin 500 mg, injection of Clindamycin 100 mg, Aenasoff lotion, ointment Momate, syrup Zincovit, fluorometholone eye drops and hypromellose opthalmic solution usp.

**Figure 1:**
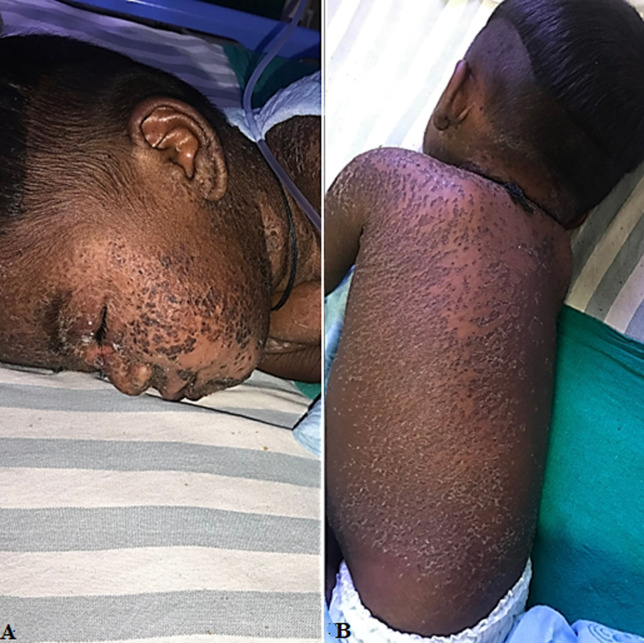
A) lesions on cheeks; B) lesions on the back

